# Ablation of Toll-like receptor 9 attenuates myocardial ischemia/reperfusion injury in mice

**DOI:** 10.1016/j.bbrc.2019.05.150

**Published:** 2019-07-30

**Authors:** Rika Kitazume-Taneike, Manabu Taneike, Shigemiki Omiya, Tomofumi Misaka, Kazuhiko Nishida, Osamu Yamaguchi, Shizuo Akira, Michael J. Shattock, Yasushi Sakata, Kinya Otsu

**Affiliations:** a The School of Cardiovascular Medicine and Sciences, King's College London British Heart Foundation Centre of Research Excellence, The James Black Centre, 125 Coldharbour Lane, London, SE5 9NU, United Kingdom; b Department of Cardiovascular Medicine, Graduate School of Medicine, Osaka University, 2-2 Yamadaoka, Suita, Osaka, 565-0871, Japan; c Laboratory of Host Defense, World Premier International Immunology Frontier Research Center, Osaka University, 2nd Fl. IFReC Research Building, 3-1 Yamadaoka, Suita, Osaka, 565-0871, Japan; d Department of Host Defense, Research Institute for Microbial Diseases, Osaka University, 3-1 Yamadaoka, Suita, Osaka, 565-0871, Japan

**Keywords:** Toll-like receptor 9, Myocardial ischemia/reperfusion injury, Langendorff-perfused mouse heart, Inflammation, Mitochondrial DNA

## Abstract

In myocardial ischemia/reperfusion injury, the innate immune and subsequent inflammatory responses play a crucial role in the extension of myocardial damage. Toll-like receptor 9 (TLR9) is a critical receptor for recognizing unmethylated CpG motifs that mitochondria contain in their DNA, and induces inflammatory responses. The aim of this study was to elucidate the role of TLR9 in myocardial ischemia/reperfusion injury.

Isolated hearts from TLR9-deficient and control wild-type mice were subjected to 35 min of global ischemia, followed by 60 min of reperfusion with Langendorff apparatus. Furthermore, wild-type mouse hearts were infused with DNase I and subjected to ischemia/reperfusion.

Ablation of TLR9-mediated signaling pathway attenuates myocardial ischemia/reperfusion injury and inflammatory responses, and digestion of extracellular mitochondrial DNA released from the infarct heart partially improved myocardial ischemia/reperfusion injury with no effect on inflammatory responses. TLR9 could be a therapeutic target to reduce myocardial ischemia/reperfusion injury.

## Highlights

• TLR9 ablation attenuates myocardial ischemia/reperfusion injury and inflammation.
• DNase I infusion partially improves myocardial ischemia/reperfusion injury.
• DNase I infusion doesn't attenuate inflammation after myocardial ischemia/reperfusion.

## 1 Introduction

Coronary heart disease is the leading cause of death worldwide. After myocardial infarction, myocardial reperfusion with primary percutaneous coronary intervention (PCI) or thrombolytic therapy is the most effective strategy to reduce infarct size and improve the clinical outcome [1], while it also initiates myocardial injury and paradoxically reduces the beneficial effect of reperfusion therapies. The pathogenesis of this phenomenon, termed myocardial ischemia/reperfusion (I/R) injury, is multifactorial [1]. The innate immune and subsequent inflammatory responses play a critical role in the extension of myocardial damage after myocardial I/R [2]. However, the molecular mechanisms responsible for initiating sterile inflammatory responses during myocardial I/R injury have not been fully elucidated.

Toll-like receptors (TLRs) were receptors for exogenous pathogens, initiating inflammation by immune cells [3]. Among TLRs, TLR9 is the only receptor which detects unmethylated CpG motifs in DNA [[3], [4], [5]], and is located intracellularly in endosomes and endoplasmic reticulum [3,4]. The activation of TLR9 induces the production of inflammatory cytokines such as tumor necrosis factor-α (TNFα), interleukin-6 (IL-6), interleukin-1β (IL-1β) and type1 interferons (IFNs) such as IFNβ. TLR9 is expressed in immune cells as well as non-immune cells such as cardiomyocytes [6].

Mitochondria contain unmethylated CpG motifs in their DNA. Damaged mitochondria in response to hemodynamic stress are degraded by autophagy-lysosomal system. We have reported that DNase II, expressed in lysosomes, degrades mitochondrial DNA (mtDNA) in autolysosomes, and incompletely digested mtDNA binds to TLR9 and induces inflammatory responses in pressure overloaded cardiomyocytes and heart failure [6].

The innate immune system in tissue injury is induced by endogenous damage-associated molecular patterns (DAMPs) released from damaged cells and contributes to sterile inflammation in injured tissues [3]. mtDNA is released from damaged cells and causes inflammatory responses to tissue injury as a DAMP [5]. It has been reported that focal myocardial necrosis leads to mtDNA release into the circulation in patients with myocardial infarction receiving PCI [7].

Thus, it was hypothesized that mtDNA released from necrotic cardiomyocytes due to myocardial I/R activates TLR9 to induce inflammation and cardiac injury. Many types of cells are involved in the development of inflammation in the heart, namely cardiomyocytes, inflammatory cells and fibroblasts. This study examined the effect of *Tlr9* ablation on myocardial I/R injury in isolated mouse hearts to exclude the involvement of circulating inflammatory cells.

## 2 Materials and methods

### 2.1 Animals

All *ex vivo* procedures in this study were carried out in accordance with the ARRIVE guidelines, the Guidance on the operation of the Animals (Scientific Procedures) Act, 1986 (UK Home Office) and EU Directive 2010/63/EU for animal experiments, and King's College London Ethical Review Process Committee and UK Home Office (Project Licence No. PPL70/7260 and 70/8889) approved the experimental protocols. Age-matched (8–11 week-old) male control C57BL/6 wild-type (WT) mice and TLR9-deficient (TLR9KO) mice with a C57BL/6 background were used in this study [6]. All mice received humane care.

### 2.2 Myocardial ischemia/reperfusion injury protocol

Isolated mouse hearts were Langendorff-perfused as previously described [8,9]. In brief, mice were anesthetized with pentobarbitone sodium and sodium heparin (140 mg/kg body weight and 200 IU, respectively, intraperitoneally). Hearts were rapidly excised and the aorta was cannulated. The hearts were then perfused with oxygenated (95% O_2_ + 5% CO_2_) Krebs-Henseleit (K-H) buffer at 37 °C (pH7.4). The K-H buffer contained 118.5 mM NaCl, 4.7 mM KCl, 1.2 mM KH_2_PO_4_, 25 mM NaHCO_3_, 1.2 mM MgSO_4_, 1.4 mM CaCl_2_, 11 mM glucose and 2.0 mM sodium pyruvate. Perfusion was in the non-recirculating Langendorff mode at a constant pressure (80 mmHg) and hearts were paced at 540 bpm. A left atrial resection was performed before insertion of a water-filled balloon through the left atrium into the left ventricle. Left ventricular pressure measurements were performed using the balloon inflated to give an end-diastolic pressure (EDP) of 5 − 10 mmHg. The EDP was adjusted to 5–10 mmHg at 5 min before ischemia. Langendorff-perfused hearts were stabilized for 20 min and subjected to 35 min of global ischemia, followed by 20 or 60 min of reperfusion.

### 2.3 Measurement of heart hemodynamic parameters

The following functional parameters were continuously recorded using a computer-based data acquisition system (PowerLab/8S with Chart 5 software, AD instruments, Gladstone, Australia): left ventricular systolic pressure (LVSP), left ventricular end-diastolic pressure (LVEDP), left ventricular developed pressure (LVDP, LVDP = LVSP - LVEDP), the maximal value of the first derivative of left ventricular pressure (dP/dt_max_), the minimal value of the first derivative of left ventricular pressure (dP/dt_min_), heart rate and coronary flow rate.

### 2.4 Assessment of myocardial infarct size

Infarct size was determined by 2,3,5-triphenyltetrazolium chloride (TTC) staining. TTC in phosphate buffered solution (pH 7.4) was infused into the hearts for 10 min at 60 min of reperfusion. Hearts were fixed in 10% formaldehyde, washed in phosphate buffered saline, and then sliced into 1 mm sections perpendicularly along the long axis from apex to base. Viable myocardium was stained in red, and infarcted tissue appeared white. The stained and fixed heart slices were digitally photographed and the areas of infarction were digitized using Image J software for planimetry. The infarct area (TTC-negative sites) and the entire ventricle area were measured by a blinded analyst. Ratios of the infarct area to the entire ventricle area were calculated and expressed as infarct size.

### 2.5 Determination of myocardial necrotic injury

Cellular necrotic injury was evaluated by measurements of creatine kinase (CK) released into the coronary effluent using an EnzyChrom Creatine Kinase Assay Kit (BioAssay Systems). During 60 min of reperfusion, all the coronary effluent dripping from hearts was collected continuously and CK concentrations in the effluent were measured by the Kit. CK release was calculated as the product of coronary flow (mL/min) and CK concentrations (U/mL) and normalized to heart weight (g).

### 2.6 Quantitative real-time RT-PCR

Total RNA from hearts was extracted using TRIzol Reagent (Thermo Fisher Scientific) and complementary DNA (cDNA) was synthesized using SuperScript II Reverse Transcriptase (Thermo Fisher Scientific). Real-time PCR was performed using Power SYBR Green PCR Master Mix (Thermo Fisher Scientific) and the sequences of PCR primers are shown in Supplementary Table S1. Real-time PCR standard curves were constructed using the corresponding cDNA. All data were normalized to *Gapdh* content and are expressed as fold increase over the control group.

### 2.7 Enzyme-linked immunosorbent assay (ELISA) for IL-6 and IFNβ in the coronary effluent

All the coronary effluent from hearts was collected continuously during 60 min of reperfusion, frozen in liquid nitrogen and analysed by ELISA according to the manufacturer's protocols (IL-6: M000B, IFNβ: MIFNB0, R&D Systems). Release of IL-6 and IFNβ from the heart was calculated as the product of quantity of coronary effluent (mL) during reperfusion and protein concentrations (ng/mL), and normalized to heart weight (g).

### 2.8 DNase I treatment to WT and TLR9KO hearts

DNase I (Sigma Aldrich, D4263) was diluted in K-H buffer (DNase I 10 Kunitz units per mL of K-H buffer). Langendorff-perfused hearts were stabilized for 10 min and perfused under constant pressure (80 mmHg) with oxygenated DNase I dilution buffer for 20 min, followed by 35 min of myocardial ischemia and 60 min of reperfusion. As a control buffer, Langendorff-perfused hearts were perfused with K-H buffer. During 60 min of reperfusion, all the coronary effluent dripping from WT hearts was collected continuously for CK measurement and WT hearts for measuring the mRNA expression of inflammatory cytokines were obtained at the end of the stabilization period and 60 min of reperfusion. For inactivated DNase I perfusion, DNase I diluted in K-H buffer was inactivated by heating (68 °C, 10 min) before perfusion.

### 2.9 Quantitative analysis of mtDNA in the coronary effluent

The coronary effluent from hearts was collected from 0 to 2 min of reperfusion and total DNA was isolated using the DNeasy Blood & Tissue Kit (QIAGEN) and purified by phenol-chloroform extraction and ethanol precipitation. For mtDNA, quantitative real-time PCR was performed using the TaqMan Gene Expression Master Mix (Thermo Fisher Scientific). The PCR primers for *Cox1* were purchased from Thermo Fisher Scientific (Mm04225243_g1). PCR standard curves were constructed using mtDNA isolated from mouse embryonic fibroblast (MEF) cells. The cells were homogenized and the mitochondrial fraction was isolated using sucrose density gradient methods [10]. The mtDNA from the mitochondrial fraction was extracted and purified using the QIAamp DNA Mini Kit (QIAGEN). The enrichment of the mtDNA fraction was confirmed by quantitative PCR analysis using mtDNA specific primers [11]. The amount of mtDNA was calculated as the product of coronary flow rate (mL/min) and mtDNA concentrations (ng/mL) [7].

### 2.10 Statistical analysis

Analysed results are expressed as the mean ± SEM. The difference between two groups was evaluated using a Student's *t*-test, and time-lapse data were assessed by two-way ANOVA repeated measures followed by Bonferroni's post hoc test. GraphPad Prism 5.0 was used for data analyses. A value of P < 0.05 was considered statistically significant.

## 3 Results

### 3.1 Effect of *Tlr9* ablation on myocardial function during I/R

**Fig. 1:**
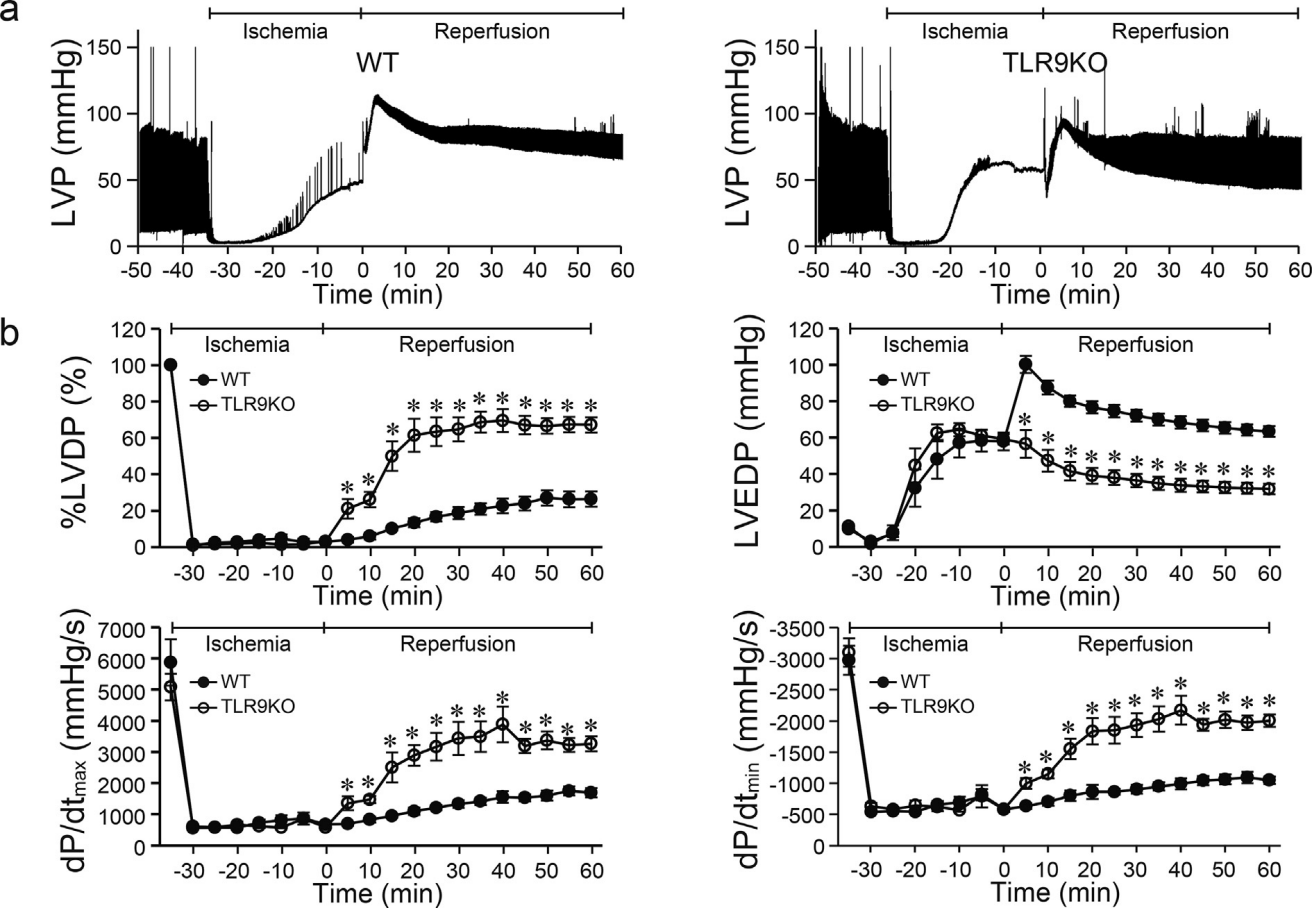
Cardiac function during myocardial ischemia/reperfusion in Langendorff-perfused control C57BL/6 (WT) and TLR9-deficient (TLR9KO) mouse hearts. **a** Representative left ventricular pressure (LVP) data. **b** Percent left ventricular developed pressure (%LVDP), left ventricular end-diastolic pressure (LVEDP), the maximal value of the first derivative of left ventricular pressure (dP/dt_max_), the minimal value of the first derivative of left ventricular pressure (dP/dt_min_). Closed circles indicate WT, open circles TLR9KO. Values represent the mean ± SEM of data from n = 6 per group. ^∗^P < 0.05 vs WT at the corresponding time point.

To investigate the role of TLR9 in cardiomyocytes in myocardial I/R, global ischemia and reperfusion was performed to isolated hearts from TLR9KO and control WT mice. Cardiac functional parameters such as LVDP, LVEDP, dP/dt_max_ and dP/dt_min_ (Fig. 1a–b) and coronary flow rate were not different at the end of the stabilization period between TLR9KO and WT hearts [coronary flow rate (mL/min), WT: 2.7 ± 0.1 vs TLR9KO: 2.8 ± 0.1, n = 6 per group]. After 35 min of ischemia, LVDP, dP/dt_max_ and dP/dt_min_ decreased, and LVEDP increased in both groups compared to those at the end of the stabilization period. However, there were no differences in LVDP, LVEDP, dP/dt_max_ or dP/dt_min_ at the end of ischemia between both groups. During reperfusion, LVDP, LVEDP, dP/dt_max_ and dP/dt_min_ recovered in both groups. However, at 5 min of reperfusion, TLR9KO hearts showed dramatic recovery of LVDP, LVEDP, dP/dt_max_ and dP/dt_min_ compared to WT hearts. The significant improvements in those parameters in TLR9KO hearts continued until 60 min of reperfusion. These results suggest that *Tlr9* ablation had no effect on cardiac dysfunction caused by ischemia, but protected cardiac function against I/R injury.

### 3.2 Effect of *Tlr9* ablation on myocardial necrosis caused by I/R

**Fig. 2:**
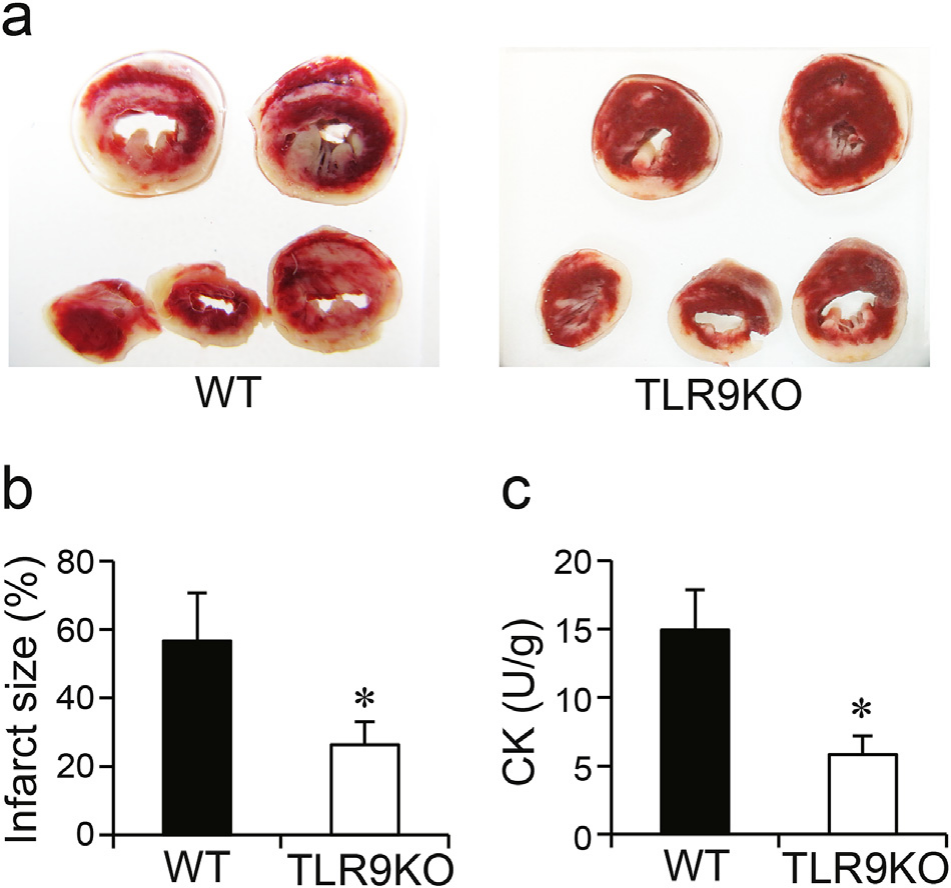
Assessment of myocardial necrosis after myocardial ischemia/reperfusion. **a** Representative images of 2,3,5-triphenyltetrazolium chloride (TTC) stained mouse heart sections. **b** Infarct size measured by TTC staining. **c** Creatine kinase (CK) release into the coronary effluent. Values represent the mean ± SEM of data from n = 6 per group. ^∗^P < 0.05 vs WT.

To examine the effect of TLR9 signaling on myocardial necrosis, infarct size was measured using TTC staining at 60 min of reperfusion (Fig. 2a and b). Infarct size in TLR9KO hearts was significantly smaller than that in WT hearts after I/R. CK release into the coronary effluent during reperfusion was measured using a CK Assay Kit (Fig. 2c). The level of CK release into the effluent in TLR9KO hearts was significantly lower than that in WT after I/R. These results indicate that greater recovery of cardiac function after I/R in TLR9KO mice was associated with myocardial necrosis.

### 3.3 Effect of *Tlr9* ablation on the inflammatory cytokine during I/R

**Fig. 3:**
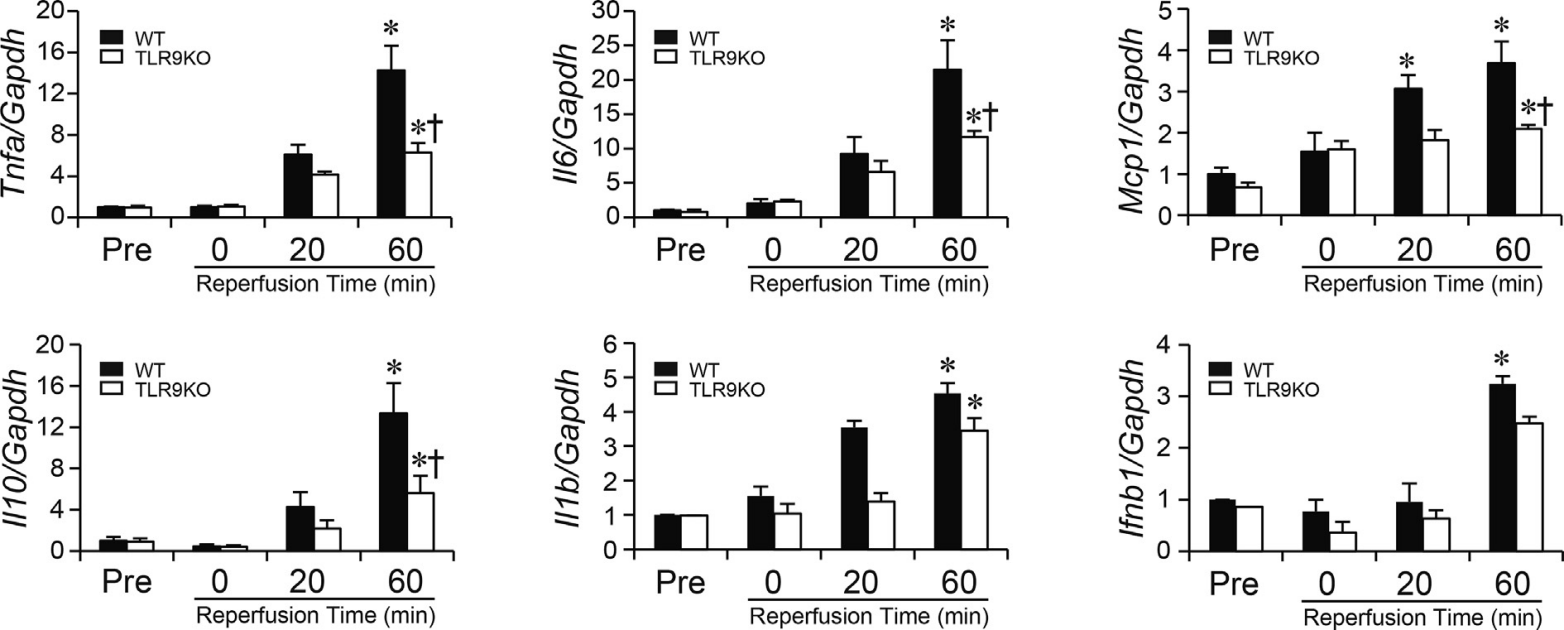
The mRNA expression levels of inflammatory cytokines derived from myocardium during myocardial ischemia/reperfusion. The mRNA levels of *Tnfa*, *Il6*, *Mcp1, Il10, Il1b* and *Ifnb1* were normalized to *Gapdh* and are shown as fold increase over levels in the control C57BL/6 (WT) group at the end of stabilization period (Pre). Closed bars indicate WT, open bars TLR9-deficient (TLR9KO) hearts. Values represent the mean ± SEM of data from n = 4–12 per group. ^∗^P < 0.05 vs the corresponding group at the end of stabilization period (Pre), ^†^P < 0.05 vs WT at the corresponding time point.

To evaluate the role of TLR9 on inflammatory responses during I/R, mRNA levels of inflammatory cytokines at the end of the stabilization period (Pre) and ischemia, and at 20 and 60 min after reperfusion were examined (Fig. 3). At the end of stabilization and ischemia, there were no significant differences in mRNA levels of inflammatory cytokines such as *Tnfa*, *Il6*, *Mcp1*, *Il10*, *Il1b* or *Ifnb1* between TLR9KO and WT hearts. However, mRNA levels of *Tnfa*, *Il6* and *Mcp1* were elevated at 60 min of reperfusion compared to those at the end of stabilization period in both TLR9KO and WT hearts. The mRNA levels were lower in TLR9KO hearts than those in WT hearts. *Il10*, as a potent anti-inflammatory cytokine, showed a similar pattern to that of *Tnfa* or *Il6*. Although mRNA levels of *Il1b* in both TLR9KO and WT hearts and *Ifnb1* in WT hearts were elevated at 60 min of reperfusion, there were no significant differences between both groups. The levels of *Ifna4* and *Ifng* mRNA expressions in the hearts were under the detectable limit during I/R (data not shown). These results suggest that ablation of *Tlr9* attenuated the inflammatory response in I/R.

Furthermore, the protein levels of IL-6 and IFNβ in the coronary effluent collected during reperfusion were measured using ELISA (Supplementary Fig. S1). The quantity of IL-6 was significantly suppressed in the effluent from TLR9KO hearts compared to WT. On the other hand, IFNβ was not detected (data not shown).

### 3.4 Effect of DNase I treatment on cardiac injury and inflammatory responses during I/R

**Fig. 4:**
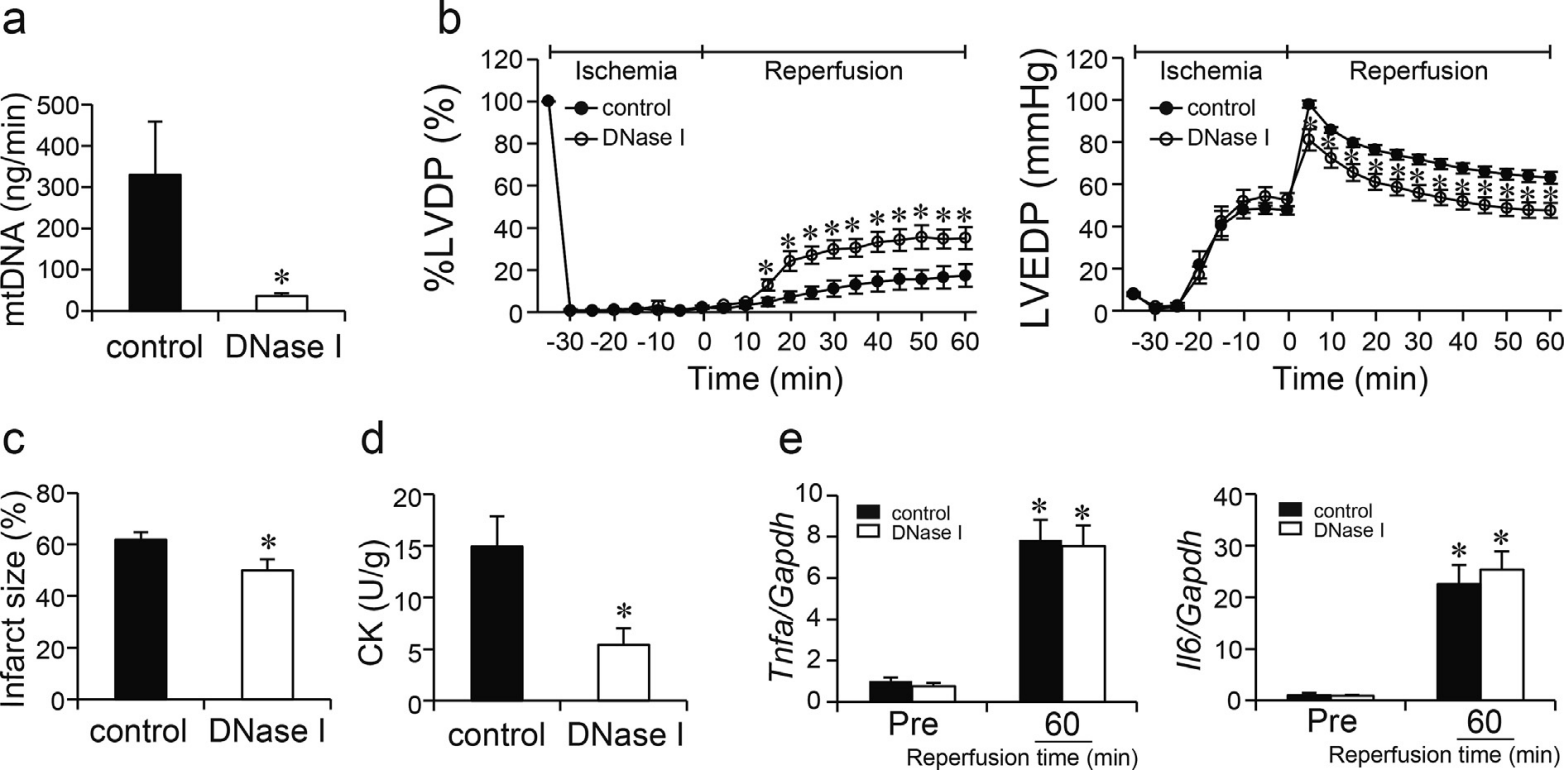
Effect of DNase I treatment during ischemia/reperfusion on Langendorff-perfused control C57BL/6 hearts in myocardial ischemia/reperfusion injury. **a** Mitochondrial DNA (mtDNA) release into the coronary effluent. Values represent the mean ± SEM of data from n = 6 per group. ^∗^P < 0.05 vs control. **b** Percent left ventricular developed pressure (%LVDP) and left ventricular end-diastolic pressure (LVEDP). Closed circles indicate control, open circles DNase I perfused hearts. Values represent the mean ± SEM of data from n = 5 per group. ^∗^P < 0.05 vs control at the corresponding time point. **c** Infarct size measured by TTC staining. Values represent the mean ± SEM of data from n = 6 per group. ^∗^P < 0.05 vs control. **d** Creatine kinase (CK) release in the coronary effluent. Values represent the mean ± SEM of data from n = 6 per group. ^∗^P < 0.05 vs control. **e** The mRNA expression levels of inflammatory cytokines derived from the myocardium during myocardial ischemia/reperfusion. The mRNA levels of *Tnfa* and *Il6* were normalized to *Gapdh* and are shown as fold increase over levels in the control group at the end of stabilization period (Pre). Closed bars indicate control, open bars DNase I perfused hearts. Values represent the mean ± SEM of data from n = 12 per group. ^∗^P < 0.05 vs the corresponding group at Pre.

To elucidate the contribution of mtDNA released from necrotic cardiomyocytes in the TLR9 signaling pathway, DNase I was perfused to hearts from WT mice during I/R. First, levels of *Cox1* DNA, derived from mtDNA, were measured in the coronary effluent to examine whether DNase I perfusion can degrade extracellular mtDNA. DNase I perfusion to isolated WT hearts decreased the level of mtDNA release into the effluent just after the beginning of reperfusion (Fig. 4a).

At the end of the stabilization period and 35 min of ischemia, LVDP, LVEDP (Fig. 4b), dP/dt_max_ and dP/dt_min_ (data not shown) were not different between DNase I perfused and control hearts. Coronary flow rate at the end of the stabilization period were not different between both groups, either [coronary flow rate (mL/min), control: 2.5 ± 0.2 vs DNase I perfused heart: 2.1 ± 0.2, n = 5 per group)]. However, during reperfusion, DNase I perfused hearts showed improvement of LVDP and LVEDP compared to control hearts, which was evident from 15 min for LVDP, and 5 min for LVEDP after reperfusion. The partial improvement of cardiac dysfunction was observed until the end of 60 min of reperfusion [dP/dt_max_ (mmHg/s) 1085 ± 222 vs 1791 ± 168; dP/dt_min_ (mmHg/s), −935 ± 164 vs −1362 ± 87, control vs DNase I perfused heart, P < 0.05, n = 5 per group]. As a control buffer for DNase I perfusion study, WT hearts were also perfused with inactivated DNase I. There was no difference in cardiac function between inactivated DNase I and control K-H buffer perfused hearts (Supplementary Fig. S2). DNase I perfused WT hearts showed significantly smaller infarct size than WT hearts perfused with control buffer (Fig. 4c). The level of CK release into the effluent was significantly lower in DNase I perfused hearts than that in control during reperfusion (Fig. 4d).

Although the levels of mRNA expression of *Tnfa* and *Il6* (Fig. 4e) as well as *Mcp1*, *Il10*, *Il1b* and *Ifnb1* (data not shown) in the hearts were elevated at 60 min of reperfusion in both groups, there were no significant differences in the mRNA expression levels between control and DNase I perfused hearts. This suggests that digestion of mtDNA released from the necrotic myocardium induced partial recovery of cardiac dysfunction and myocardial necrosis during I/R, but had no effect on the inflammatory responses.

Furthermore, DNase I was perfused to isolated hearts from TLR9KO mice during I/R. There was no difference in cardiac function between TLR9KO hearts perfused with DNase I and TLR9KO hearts perfused with control K-H buffer (Supplementary Fig. S3). This result suggests that non-TLR9 receptors were not main players mediating mtDNA signaling in this model.

## 4 Discussion

This study showed that ablation of the myocardial TLR9 signaling pathway attenuated inflammatory responses and myocardial I/R injury and that mtDNA released from necrotic cardiomyocytes could activate TLR9.

The role of TLR9 in myocardial I/R injury has been investigated using an *in vivo* model, where TLR9 was activated by the administration of unmethylated CpG oligonucleotides. Activation of TLR9 prior to I/R improved cardiac function and reduced infarct size [12,13], while TLR9 activation upon onset of ischemia showed no effect on infarct size despite causing myocardial inflammation [14]. However, *Tlr9* ablation reduced infarct size in I/R hearts [13]. In the present study, global ischemia was performed in an *ex vivo* model to eliminate the contribution of circulating immune cells, showing that ablation of *Tlr9* had no effect on cardiac function during ischemia, but improved recovery of both systolic and diastolic cardiac function during reperfusion. The protection was initiated in the early phase of reperfusion, indicating that TLR9 activation in the heart during reperfusion is detrimental to the heart. The reduction in the infarct size and CK release into the coronary effluent suggests that the detrimental effect of TLR9 signaling in I/R hearts may be caused by the induction of cardiomyocyte necrotic death.

For the first time, we showed that ablation of TLR9 in the heart attenuates inflammatory responses in I/R without the involvement of circulating immune cells in an *ex vivo* study. Inflammatory cytokines such as TNFα and IL-6 are reported to depress myocardial function in an *ex vivo* crystalloid-superfused papillary muscle preparation [15]. Initially, it was hypothesized that inflammatory cytokines are main contributors to cardiac dysfunction in I/R. However, recovery of cardiac function in TLR9KO hearts was detected in the early phase of reperfusion before downregulation of inflammatory cytokines was detected. In addition, DNase I treatment attenuated I/R injury but showed no effect on inflammatory responses. A previously reported study revealed inflammation-independent roles of TLR9 in cardiomyocytes [16]. Shintani et al. reported that TLR9 stimulation reduces energy substrates and increases the AMP/ATP ratio, subsequently activating AMP-dependent kinase, leading to increase tolerance against hypoxia in cardiomyocytes without inducing the canonical inflammatory responses. We have previously reported that TLR9 prevents cardiac rupture after myocardial infarction by promoting proliferation and differentiation of cardiac fibroblasts [17]. It is possible that cardiac dysfunction in I/R may be not only induced by TLR9-mediated inflammatory cytokine activation but also mediated through the inflammation-independent TLR9 signaling pathway.

Extracellular mtDNA can be internalized into cardiomyocytes inducing the TLR9 signaling and cell death [18]. DNase I treatment reduced infarct size in myocardial I/R open-chest or isolated perfused rat hearts [19]. Furthermore, the combination of DNase I and mitochondria-targeted endonuclease III, which would maintain mitochondrial integrity in the ischemic cardiomyocytes, can produce additive protection against myocardial I/R injury [19]. On the other hand, we found that intracellular mtDNA escaping from autophagic degradation can cell-autonomously activate TLR9 and induce the inflammatory response in pressure overloaded cardiomyocytes [6]. In this study, it was examined whether the degradation of extracellular mtDNA can rescue the cardiac phenotypes and inhibit the inflammatory responses in I/R injury by perfusing DNase I into the heart. The inflammatory responses, which were inhibited by *Tlr9* ablation, were not affected by DNase I perfusion in I/R hearts. DNase I is supposed to degrade mainly extracellular mtDNA and prevent extracellular mtDNA from being internalized into cardiomyocytes. We do not know the exact reason why mtDNA internalized from extracellular space and that generated cell-autonomously inside the cell have apparently different outcome following TLR9 activation. TLR9 locates intracellularly in endosomes and endoplasmic reticulum [3,4] as well as autolysomes [6]. Each type of mtDNA might activate distinct TLR9, which has different subcellular localization and function.

This study revealed that TLR9 in the heart may be a critical contributor to the final infarct size and could be a therapeutic target in myocardial I/R injury. TLR9 has a wide variety of functions in cardiac disease, so further research is needed to determine the risks and benefits of TLR9 as a therapeutic target and to access the method of application.

## Conflicts of interest

The authors have no conflict of interest to declare.

## Appendix A Supplementary data

Application Application

Application Application

Application Application

Application Application
