# Supplementary material for: Ablation of Toll-like receptor 9 attenuates myocardial ischemia/reperfusion injury in mice
Source: Biochem Biophys Res Commun. 2019 Jul 30;515(3):442–7. doi: 10.1016/j.bbrc.2019.05.150 (PMC6590932; doi:10.1016/j.bbrc.2019.05.150)
Supplement: Application [file mmc2.docx]

Supplementary Table S1. Quantitative real-time PCR primers

| Gene | Direction | Primer Sequence (5' to 3') |
| --- | --- | --- |
| *Tnfa* | forward | TCCCAGGTTCTCTTCAAGGGA |
|  | reverse | GGTGAGGAGCACGTAGTCGG |
| *Il6* | forward | ACAACCACGGCCTTCCCTACTT |
|  | reverse | CACGATTTCCCAGAGAACATGTG |
| *Mcp1* | forward | CCACTCACCTGCTGCTACTCAT |
|  | reverse | TGGTGATCCTCTTGTAGCTCTCC |
| *Il10* | forward | GTGAAGACTTTCTTTCAAACAAAG |
|  | reverse | CTGCTCCACTGCCTTGCTCTTATT |
| *Il1b* | forward | AAGAGCTTCAGGCAGGCAGTATCA |
|  | reverse | TAATGGGAACGTCACACACCAGCA |
| *Ifnb1* | forward | CTTCTCCGTCATCTCCATAGGG |
|  | reverse | CACAGCCCTCTCCATCAACT |
| *Ifna4* | forward | CTGCTGGCTGTGAGGACATA |
|  | reverse | TTGCTCAAGATTGCTGAAACA |
| *Ifng* | forward | CGGCACAGTCATTGAAAGCCTA |
|  | reverse | GTTGCTGATGGCCTGATTGTC |
| *Gapdh* | forward | ATGACAACTTTGTCAAGCTCATTT |
|  | reverse | GGTCCACCACCCTGTTGCT |
