# Supplementary material for: Ablation of Toll-like receptor 9 attenuates myocardial ischemia/reperfusion injury in mice
Source: Biochem Biophys Res Commun. 2019 Jul 30;515(3):442–7. doi: 10.1016/j.bbrc.2019.05.150 (PMC6590932; doi:10.1016/j.bbrc.2019.05.150)
Supplement: Application [file mmc3.pdf]

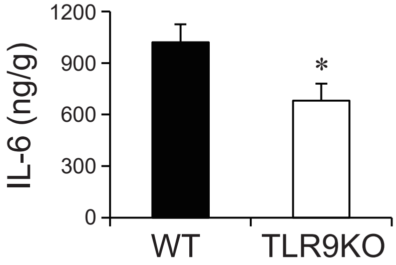

Supplementary Figure S1

The level of IL-6 protein in the coronary effluent collected from WT and TLR9KO hearts during reperfusion. Values represent the mean ± SEM of data from n = 6 per group. *$P < 0.05$ vs WT.
