# Supplementary material for: Ablation of Toll-like receptor 9 attenuates myocardial ischemia/reperfusion injury in mice
Source: Biochem Biophys Res Commun. 2019 Jul 30;515(3):442–7. doi: 10.1016/j.bbrc.2019.05.150 (PMC6590932; doi:10.1016/j.bbrc.2019.05.150)
Supplement: Application [file mmc4.pdf]

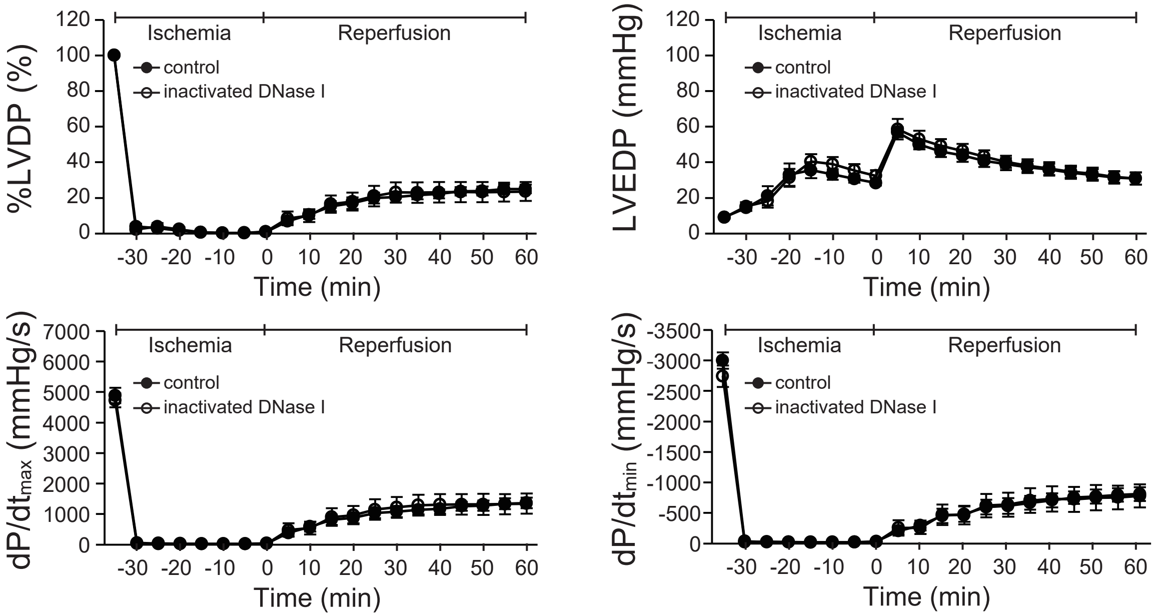

Supplementary Figure S2

Effect of inactivated DNase I perfusion on cardiac function of Langendorff-perfused WT hearts in myocardial ischemia/reperfusion injury. Percent left ventricular developed pressure (%LVDP), left ventricular end-diastolic pressure (LVEDP), the maximal value of the first derivative of left ventricular pressure (dP/dt_{max}), the minimal value of the first derivative of left ventricular pressure (dP/dt_{min}). Closed circles indicate WT control, open circles inactivated DNase I perfused WT hearts. Values represent the mean ± SEM of data from n = 6 per group.
