# Supplementary material for: Ablation of Toll-like receptor 9 attenuates myocardial ischemia/reperfusion injury in mice
Source: Biochem Biophys Res Commun. 2019 Jul 30;515(3):442–7. doi: 10.1016/j.bbrc.2019.05.150 (PMC6590932; doi:10.1016/j.bbrc.2019.05.150)
Supplement: Application [file mmc5.pdf]

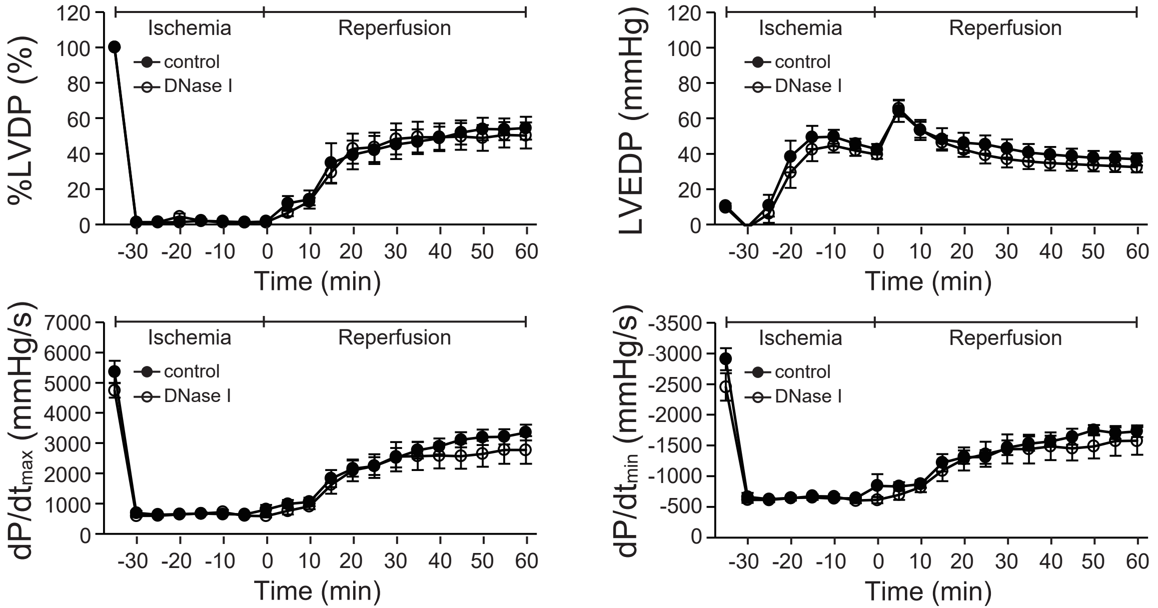

Supplementary Figure S3

Effect of DNase I treatment on cardiac function of Langendorff-perfused TLR9KO mouse hearts in myocardial ischemia/reperfusion injury. Percent left ventricular developed pressure (%LVDP) and left ventricular end-diastolic pressure (LVEDP), the maximal value of the first derivative of left ventricular pressure (dP/dt_{max}), the minimal value of the first derivative of left ventricular pressure (dP/dt_{min}). Closed circles indicate control TLR9KO, open circles DNase I perfused TLR9KO hearts. Values represent the mean ± SEM of data from n = 5 per group.
